# Nano-sized warriors: zinc chromium vanadate nanoparticles as a dual solution for eradicating waterborne enterobacteriaceae and fighting cancer

**DOI:** 10.3389/fphar.2023.1213824

**Published:** 2023-07-13

**Authors:** Suriya Rehman, Fatimah Alahmari, Laila Aldossary, Maryam Alhout, Suhailah S. Aljameel, Syed Mehmood Ali, Jamal S. M. Sabir, Firdos Alam Khan, Irfan A. Rather

**Affiliations:** ^1^ Department of Epidemic Diseases Research, Institute for Research and Medical Consultations (IRMC), Imam Abdulrahman Bin Faisal University, Dammam, Saudi Arabia; ^2^ Department of Nanomedicine Research, Institute for Research and Medical Consultations (IRMC), Imam Abdulrahman Bin Faisal University, Dammam, Saudi Arabia; ^3^ Summer Research Program, Institute for Research and Medical Consultations (IRMC), Department of Environmental Sciences, College of Science, Imam Abdulrahman Bin Faisal University, Dammam, Saudi Arabia; ^4^ Department of Chemistry, College of Science, Imam Abdulrahman Bin Faisal University, Dammam, Saudi Arabia; ^5^ Department of Biomedical Engineering, College of Engineering, Imam Abdulrahman Bin Faisal University, Dammam, Saudi Arabia; ^6^ Department of Biological Science, Faulty of Science, King Abdulaziz University, Jeddah, Saudi Arabia; ^7^ Center of Excellence in Bionanoscience Research, King Abdulaziz University, Jeddah, Saudi Arabia; ^8^ Department of Stem Cell Research, Institute for Research and Medical Consultations (IRMC), Imam Abdulrahman Bin Faisal University, Dammam, Saudi Arabia

**Keywords:** nanomaterial, nanotherapeutics, antibacterial activity, anticancer: small molecule, zinc chromium vanadate

## Abstract

The revolution of biomedical applications has opened new avenues for nanotechnology. Zinc Chromium vanadate nanoparticles (VCrZnO4 NPs) have emerged as an up-and-coming candidate, with their exceptional physical and chemical properties setting them apart. In this study, a one-pot solvothermal method was employed to synthesize VCrZnO4 NPs, followed by a comprehensive structural and morphological analysis using a variety of techniques, including X-Ray diffraction, scanning electron microscopy, high-resolution transmission electron microscopy, Energy-dispersive X-ray, and X-ray photoelectron spectroscopy. These techniques confirmed the crystallinity of the NPs. The VCrZnO4 NPs were tested for their antibacterial activity against primary contaminants such as Enterobacteriaceae, including *Shigella flexneri*, *Salmonella cholerasis*, and *Escherichia coli*, commonly found in hospital settings, using the broth dilution technique. The results indicated a stronger antibacterial activity of VCrZnO4 NPs against *Shigella* and *Salmonella* than *E. coli.* Electron microscopy showed that the NPs caused severe damage to the bacterial cell wall and membrane, leading to cell death. In addition, the study evaluated the anticancer activities of the metal complexes *in vitro* using colorectal cancer cells (HCT-116) and cervical cancer cells (HELA), along with non-cancer cells and human embryonic kidney cells (HEK-293). A vanadium complex demonstrated efficient anticancer effects with half-inhibitory concentrations (IC_50_) of 38.50+3.50 g/mL for HCT-116 cells and 42.25+4.15 g/mL for HELA cells. This study highlights the potential of Zinc Chromium vanadate nanoparticles as promising candidates for antibacterial and anticancer applications. Various advanced characterization techniques were used to analyze the properties of nanomaterials, which may help develop more effective and safer antibacterial and anticancer agents in the future.

## 1 Introduction

In recent years, the use of metal ions in combination with organic or inorganic ligands to form complexes has gained significant attention due to their enhanced activity and potential biomedical applications. This gave the metal ion complexes far greater utility in medicine, chemical, pharmaceuticals, and agricultural industries (Kongot et al., 2020). Many studies indicated the detailed analysis and focused on the chemistry and synthesis of such metal compounds for their potential applications in the clinical treatment of various diseases, including communicable (infectious diseases) and non-communicable (cancer) diseases. As a result, recent research has shifted toward synthesizing metal complexes with potential biological properties, such as an antimicrobial and anticancer action. Recent studies have indicated that complexes of metals such as chromium, cobalt, copper, vanadium, manganese, and zinc have huge potential as antimicrobial, anticancer, antioxidant, and antidiabetic properties (Senthil Raja et al., 2012; Al-Hazmi et al., 2020). In this regard, Vanadium and chromium play crucial roles and are known for their significant bioactivities like antimicrobial, anticancer, and antiviral (Crans et al., 2018; 2019; Selman et al., 2018; Assey and Mgohamwende, 2020; Suma et al., 2020). Vanadium can exist in many oxidation states; therefore, vanadium complexes are attractive to researchers for their promising biological activities. Several reports are on synthesizing vanadium complexes with different ligands to determine their magnetic, antidiabetic, antioxidant, and catalytic properties (Crans et al., 2019; Assey and Mgohamwende, 2020). However, vanadium and chromium-based complexes’ antibacterial and anticancer activity is rarely studied (Singh and Gupta, 2008; Al-Hazmi et al., 2020).

Meanwhile, the global rise of microbial resistance to antibiotics has become a major public health concern. Bacteria have developed mechanisms to resist the effects of almost all available antibiotics, resulting in increased morbidity and mortality from life-threatening infections. This scenario presents a major challenge, particularly for hospitalized patients more susceptible to such infections (Karataş et al., 2021). In this context, nanotechnology has emerged as a potential alternative to conventional antimicrobials, as it offers unique opportunities for the development of nano products that can be utilized in the prevention, diagnosis, and targeted delivery of drugs (Shu et al., 2017; de Oliveira et al., 2020). By harnessing the properties of nanoparticles, such as their high surface area and reactivity, nanotechnology enables the creation of novel antimicrobial solutions. These nanoproducts have shown promising results in combating drug-resistant microorganisms and addressing the limitations of traditional antibiotics. Likewise, colon and cervical cancer are among the significant global health concerns, estimating 1.08 million new cases in 2020 for colon cancer alone (Sun et al., 2018; Mohanty et al., 2019). Current treatment options for cancer, including surgery, chemotherapy, radiotherapy, and immunotherapy, have limitations in effective metastasis and inhibiting tumor growth (Miller et al., 2019). Furthermore, prolonged consumption of chemotherapeutic drugs often leads to adverse side effects that could be life-threatening and painful for patients.

Consequently, there is a growing interest in developing innovative methods for controlling and treating cancer cells that are precise in their delivery and have fewer adverse effects. Nanotechnology offers promising approaches in this regard. By utilizing nanoparticles, it is possible to achieve targeted drug delivery, enhance the efficacy of cancer treatments, and minimize the side effects associated with conventional therapies (Schirrmacher, 2019).

Therefore, the convergence of nanotechnology and biomedical research has opened up new avenues for addressing the challenges posed by microbial resistance and cancer treatment. In this context, exploring metal-based nanoparticles, such as Zinc Chromium vanadate nanoparticles (VCrZnO4 NPs), holds excellent promise. VCrZnO4 NPs exceptional physical and chemical properties make them suitable candidates for antibacterial and anticancer applications (Crans et al., 2018; Assey and Mgohamwende, 2020; Suma et al., 2020). By bridging the knowledge gap and advancing our understanding of the biological activities of vanadium and chromium-based complexes, this study presents an innovative approach for synthesizing VCrZnO4 NPs using a one-pot solvothermal reaction with tetramethylammonium hydroxide (TMAOH) as both a reducing agent and a structure-directing agent. Notably, using TMAOH, uncommon in Zn metalate preparation, allows for the direct separation of the crystalline phase from the reaction solution, eliminating the need for an annealing step. Our research aims to shed light on the potential applications of VCrZnO4 NPs in combating microbial resistance and improving cancer treatment strategies. By exploring the unique properties of these nanoparticles, the study aims to contribute to the advancement of nanomedicine and bridge the knowledge gap regarding the biological activities of vanadium and chromium-based complexes in the realms of antibacterial and anticancer research. This exploration will not only enhance our understanding of the potential applications of VCrZnO4 nanoparticles but may also facilitate the advancement of nanomedicine. The findings of this study may pave the way for the development of more effective and targeted approaches in the fields of antibacterial treatments and anticancer therapies.

## 2 Materials and methods

### 2.1 Synthesis

**FIGURE 1:**
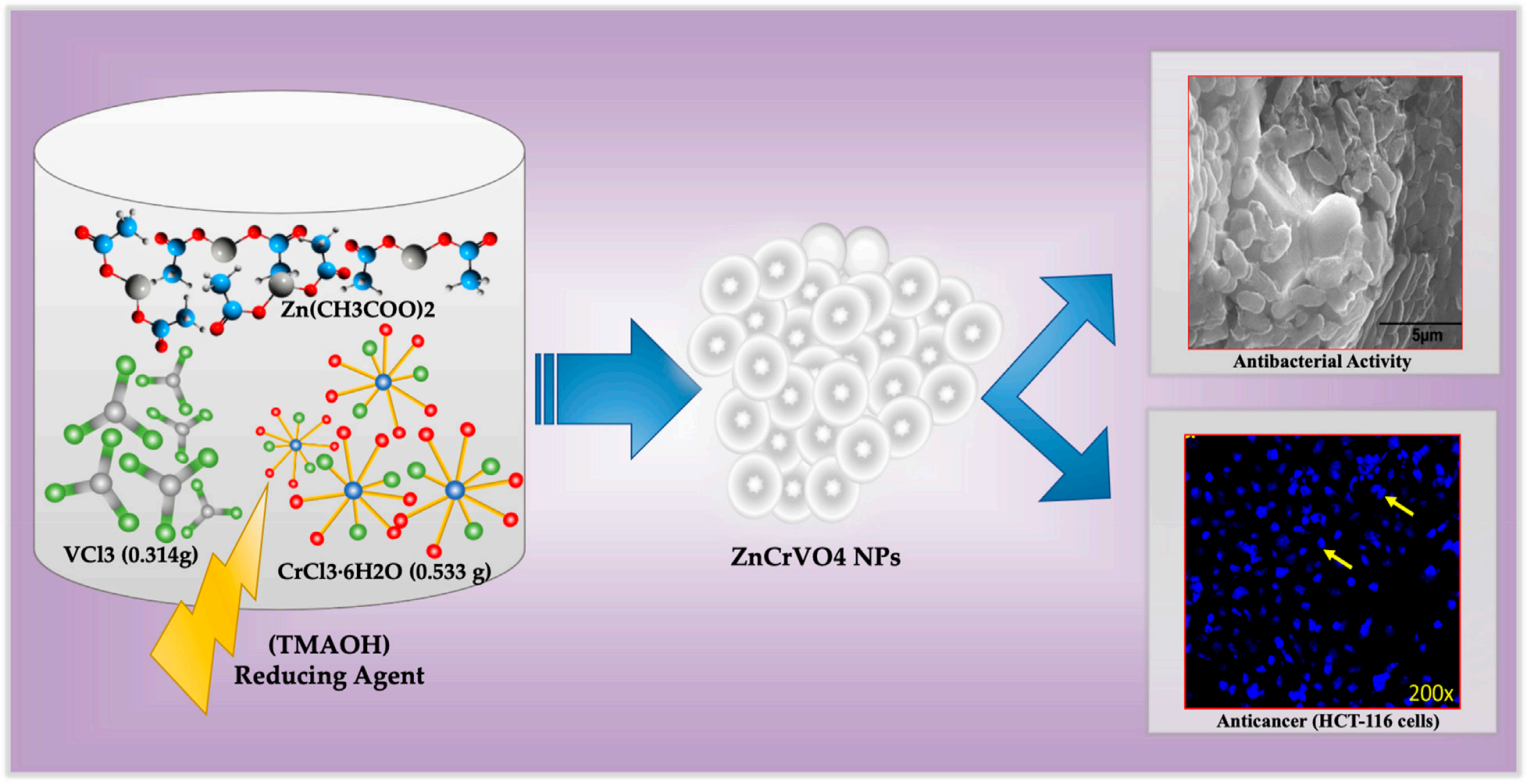
Schematic representation of the synthesis protocol for ZnCrVO4 nanoparticles (NPs) with antibacterial and anticancer activities.

ZnCrVO4 NPs were prepared by mixing 2 mmol of each CrCl3.6H2O (0.533 g), VCl3 (0.314 g), and Zn(CH3COO)2·2H2O (0.44 g), with 140 mL of DI water in a 200 mL Teflon-lined container before dropping 10 mL of tetramethylammonium hydroxide (TMAOH) solution of 25 wt% in H2O to the mixture. Then, the container was closed and tightly sealed into a stainless-steel autoclave and placed in a preheated oven at 200°C for 48 H. After cooling to room temperature, the precipitated dark grey NPs were separated from the solution by centrifuging and washing with DI water several times until a clear solution was obtained. The sample was washed with absolute ethanol, dried with diethyl ether, and placed in a vacuum oven at 80°C overnight. The yield of the prepared NPs after the washing and drying process was approximately 98%, which confirms that the starting materials reacted completely under the specified conditions. Figure 1 illustrates the synthesis protocol for ZnCrVO4 nanoparticles (NPs) with antibacterial and anticancer activities.

### 2.2 Characterization

The method employed in this study involved several characterization techniques to analyze the Zinc Chromium vanadate nanoparticles (VCrZnO4 NPs). Powder X-Ray Diffraction (PXRD) analysis was conducted using a Rigaku Benchtop Miniflex X-ray powder diffractometer equipped with a Cu Kα radiation generator. The measurement was carried out in a room, scanning the 2θ range of 20–70° with a step size of 0.05, allowing for the determination of the crystalline structure of the nanoparticles. High-resolution transmission electron microscopy (HRTEM) imaging was performed using a TEM instrument, specifically the FEI Titan 80–300 kV FEGS/TEM. This technique provided detailed images of the nanoparticles at high magnification, allowing for the examination of their morphology and structure. The HRTEM analysis was coupled with Energy-dispersive X-ray spectroscopy (EDX) for elemental mapping, enabling the identification and spatial distribution of different elements within the nanoparticles.

Furthermore, X-ray photoelectron spectroscopy (XPS) analysis was carried out using a Kratos Axis Ultra instrument, equipped with a monochromatic X-ray source (Al Kα). The XPS analysis was conducted under an extremely high vacuum (10–9 mbar) and provided valuable information about the chemical composition and surface properties of the nanoparticles. Together, these characterization techniques offered a comprehensive analysis of the structural, morphological, and elemental characteristics of the VCrZnO4 NPs, contributing to a better understanding of their properties and potential applications (Aygar et al., 2015).

### 2.3 Antibacterial studies

#### 2.3.1 Inoculum preparation

For the antibacterial studies, Gram-negative bacteria were selected as the target pathogens. Specifically, *Shigella flexneri* (ATCC12022) (Philpott et al., 2000), *Salmonella choleraesuis* (ATCC10708) (Chiu et al., 2004), and *Escherichia coli* (ATCC352118) (Kaper et al., 2004) were chosen as representative human pathogens. These bacteria were cultured in Nutrient broth (NB) at 37°C ± 2°C for an overnight incubation period (Rather et al., 2023). Subsequently, during the exponential growth phase, the bacterial cells were harvested. To prepare the cells for further antibacterial analysis, they were washed with PBS and suspended in the same buffer. The concentration of the bacterial suspension was adjusted to 1 × 10^6^ CFU/mL, ensuring a standardized inoculum size for the subsequent antibacterial assays. This standardized approach enabled accurate and consistent assessment of the antibacterial activity of the Zinc Chromium vanadate nanoparticles against these specific Gram-negative bacteria.

#### 2.3.2 Broth dilution method

The broth dilution technique was used to evaluate the Minimum Inhibitory Concentration (MIC) and Minimum Bactericidal Concentration (MBC) of the synthesized nanomaterial VCrZnO4 using the same method described elsewhere (Wijesundara et al., 2021; Rather et al., 2022a). Samples were sonicated and diluted with Mueller Hinton broth (MHB) to obtain homogenized broth suspension at 16, 8, 4, and 2 mg/mL. The adjusted inoculum was added to the prepared broth solution at 1 × 10^6^ CFU/mL. Bacteria were incubated at 37°C ± 2 °C for 18–20 h. Control was taken as untreated bacteria under the same conditions. After incubation, antibacterial activity was determined by obtaining the MIC and MBC. Briefly, the aliquot of incubated bacterial suspension was streaked onto freshly prepared MHA plates, which were then incubated at 37°C overnight. Then, the plates were observed, and the MIC was taken as the concentration of the nanomaterial at which the 70%–90% growth is inhibited, while the MBC was taken as the concentration at which no cell or less than 3 cells were found (Rehman et al., 2019).

#### 2.3.3 Morphogenesis of treated bacterial cells

Morphological changes in bacteria treated with VCrZnO4 were studied using scanning electron microscopy (SEM) (Rather et al., 2023). The representative concentration obtained as MIC was used to treat *S. flexneri, S. cholerasuis*, and *E. coli*, while untreated bacteria were used as a control. The cells were washed multiple times in PBS and then centrifuged to remove the residual medium. After that, the cells were fixed with 2.5% glutaraldehyde for 6 h, followed by fixation with 1% osmium tetroxide for 1 h. Next, the cells were dehydrated using varying concentrations of ethanol solution and again washed and suspended in PBS. Finally, the cells were placed on a stub and dried in a desiccator to complete the drying process. After gold coating, the samples were analyzed under SEM at 20 kV using a scanning electron microscope (Rehman et al., 2020).

### 2.4 Anticancer studies

#### 2.4.1 *In vitro* testing of cell viability by MTT assay

Human colorectal cancer cells were obtained from American Type Cell Culture ATCC, United States, and were used to study the viability of cancer cells mediated by nanoparticles VCrZnO4 (Alzahrani et al., 2022; Rather et al., 2022b). HEK-293 cells were also used as a control to ensure the effectiveness of the study. First, 96 well plates containing special DMEM media were seeded in a CO2 incubator and kept for 24 h. To conduct the MTT assay, the nanocomposites VCrZnO4 were added to each well containing HCT-116, HELA, and HEK-293 cells in concentrations ranging from 2.0 μg/mL to 75 μg/mL for 48 h. The control wells were not treated with nanoparticles VCrZnO4. After treatment, the cells were exposed to MTT (5.0 mg/mL) for 4 h, and the media was replaced with DMSO. The optical density (OD) of the cells was then measured at 570 nm using a Plate Reader supplied by Bio-Tek Instruments, United States. The percentage of cell viability was calculated based on OD, and the data obtained from triplicates were statistically evaluated using GraphPad Version 6.0 Prism Software United States. Mean ± standard deviation values were presented in the graph (Khan et al., 2018).

#### 2.4.2 Apoptotic DAPI staining

To examine the impact of nanocomposite VCrZnO4 on the nucleus of cancer HCT-116 and HELA cells, we stained cells with DAPI staining post-48-h treatment of nanoparticles VCrZnO4 with 60 μg/mL. In brief, cells were treated with paraformaldehyde and labeled with DAPI dye. The blue, fluorescent staining was examined under a Confocal Scanning Microscope (Zeiss, Germany) (Zeiss, Germany) (Khan et al., 2018).

## 3 Results and discussion

Structural and morphological studies of ZnVCrO_4_ NPs have been conducted using various techniques, including PXRD SEM, TEM, and XPS. These studies have provided valuable insights into the crystal structure and morphology of this compound, which are essential for understanding its potential applications in various fields.

### 3.1 Structural and morphological studies

**FIGURE 2:**
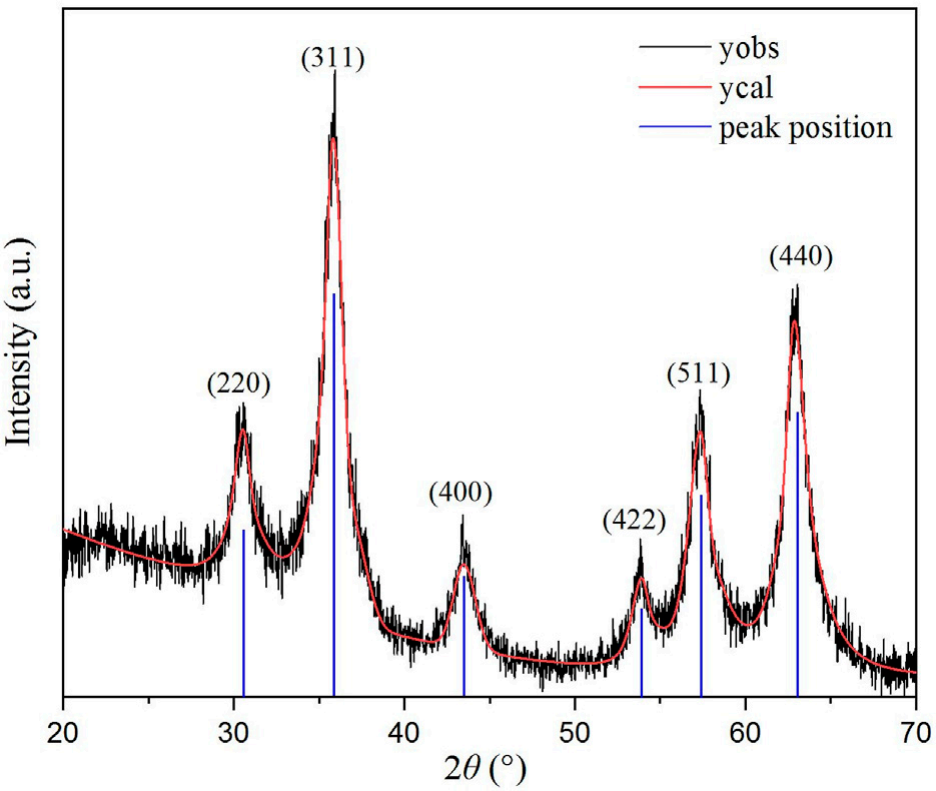
PXRD pattern for ZnCrVO_4_ NPs recorded at room temperature.

The measured PXRD pattern for ZnVCrO_4_ is displayed in Figure 2. The plot shows the presence of only one crystalline phase corresponding to a cubic close-packed spinel structure. The lattice constant *a* was calculated through the most intense peak corresponding to (311) to be *a* = 8.55 Å.

**FIGURE 3:**
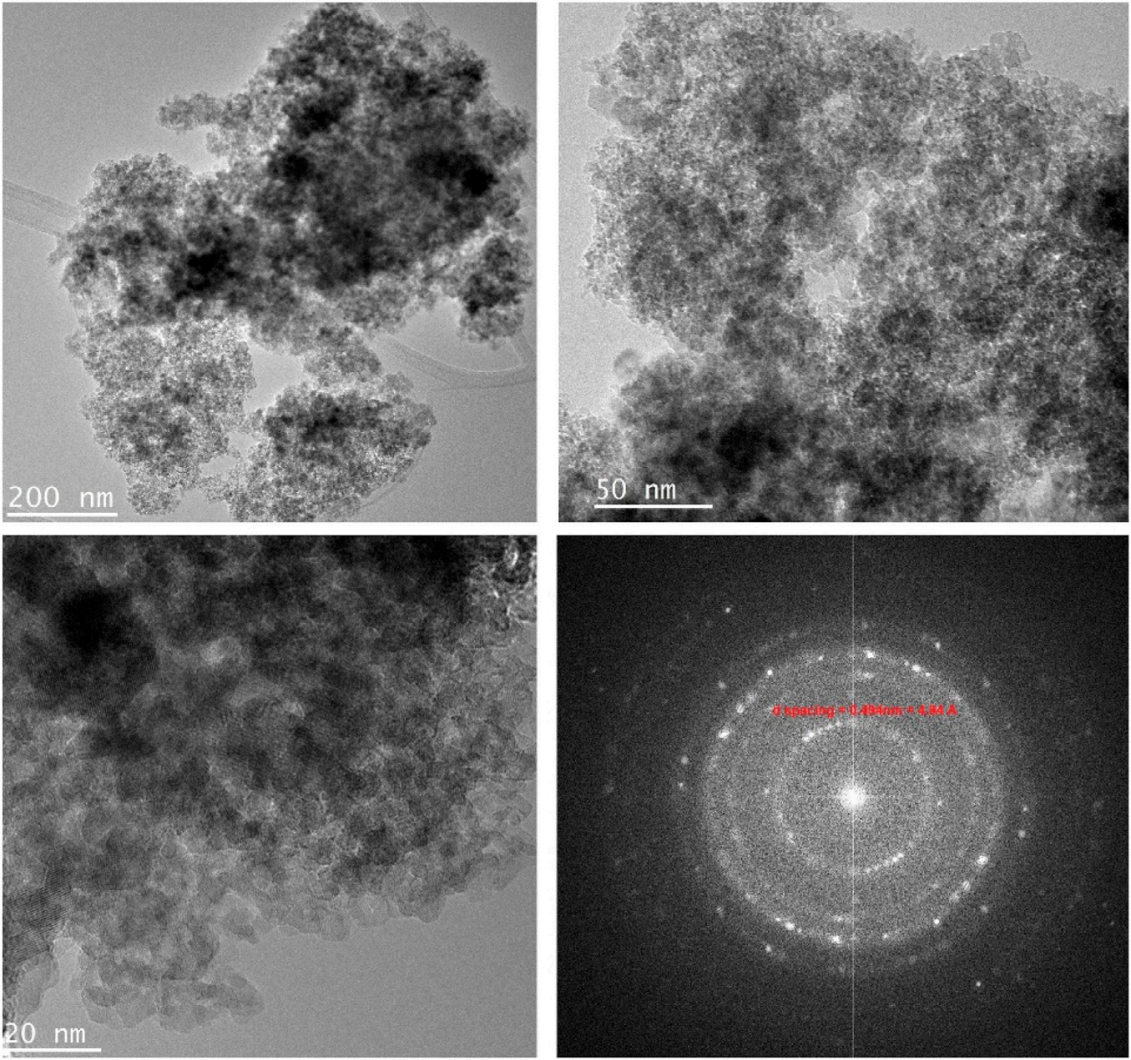
TEM images and SEAD analysis for ZnCrVO_4_ NPs.

**FIGURE 4:**
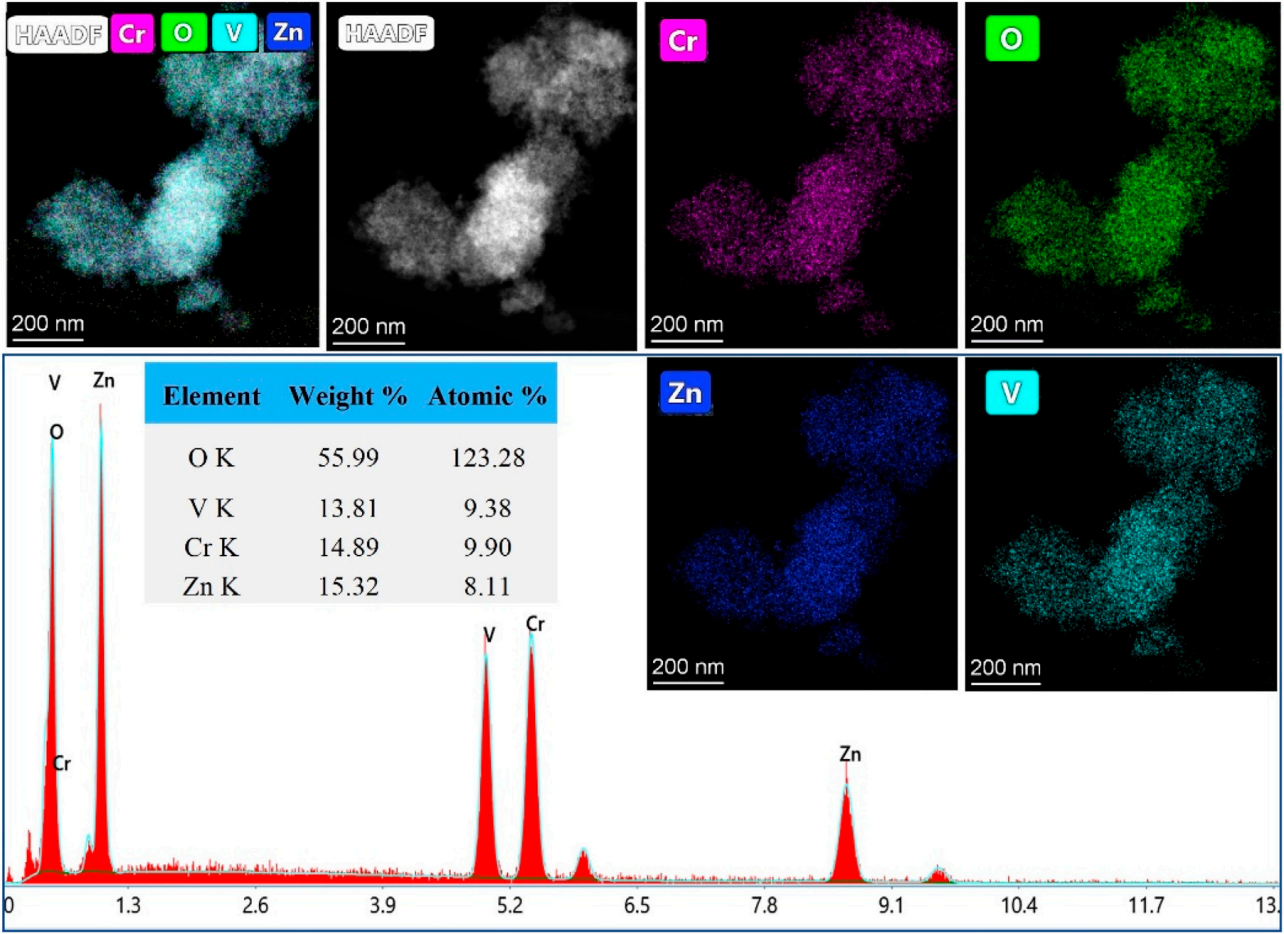
HAADF elemental mapping and EDX analysis for ZnCrVO_4_ NPs.

TEM images, captured in different resolutions, in Figure 3 show aggregation of nanoparticles with a size of ˂ 20 nm. The selected Area Electron Diffraction (SAED) has been used to confirm the crystallinity of the prepared NPs. The interplanar distance (*d*-spacing) was determined from the lattice fringes of the first diffraction ring. The calculated *d*-spacing is 4.94 Å, corresponding to (111) plan of spinel lattice which agreed well with the PXRD analysis. Figure 4 shows the elemental mapping and EDX analysis for ZnVCrO_4_ NPs, confirming the presence of the Zn, V, Cr, and O exclusively in the sample with the elemental ratio of ≈1:1:1:4, respectively.

**FIGURE 5:**
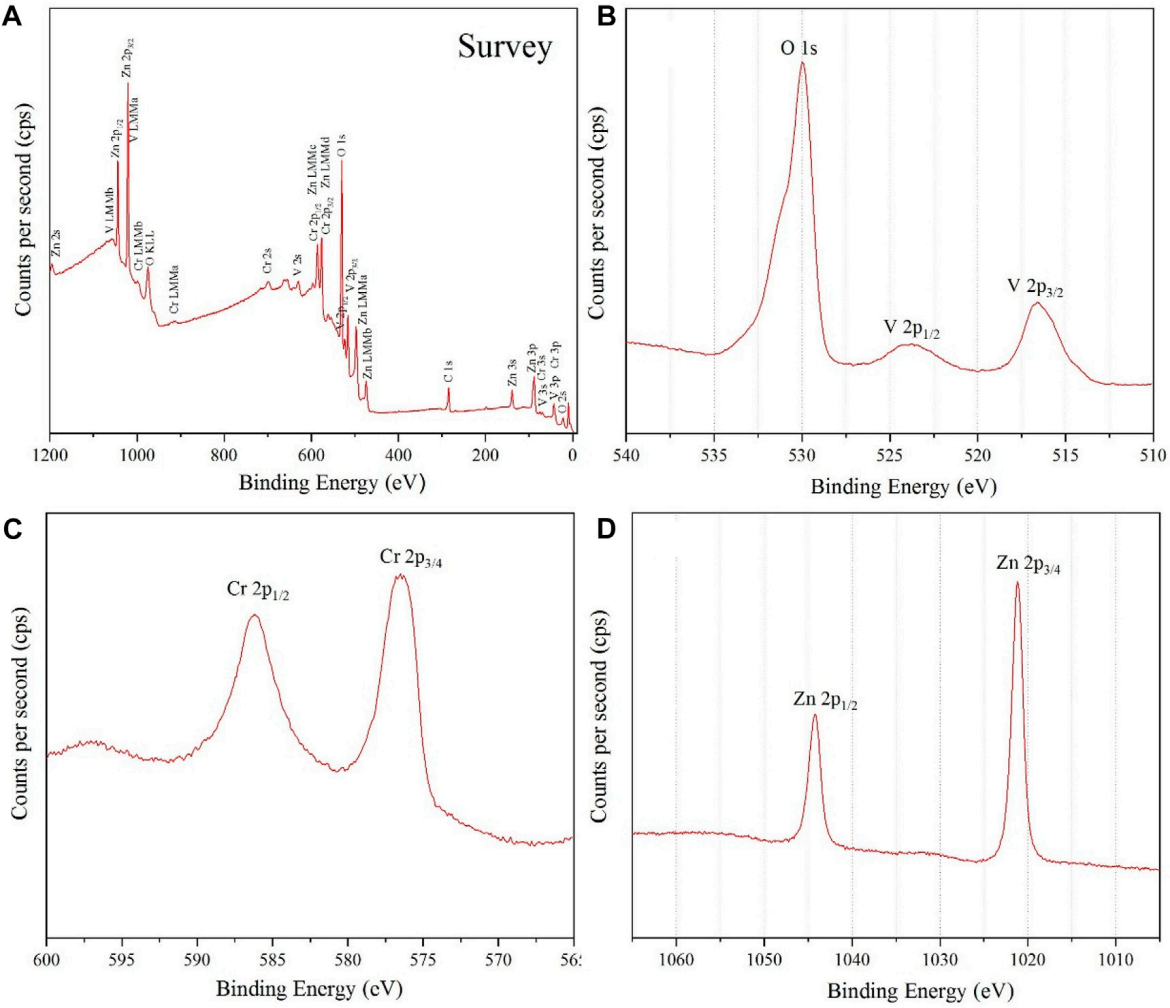
**(A)** survey analysis, **(B)** narrow V 2p and O 1s XPS spectrum, **(C)** Cr 2p XPS spectrum and **(D)** Zn 2p XPS spectrum, for ZnCrVO_4_ NPs.

For the fourth identification and confirmation of phase purity, XPS analysis was performed on the prepared NPs. A survey scan for detecting all elements in the sample is shown in Figure 5A.

The narrow spectrum in Figure 5B shows two peaks at 516.49 and 523.79 eV with a spin splitting of 7.3 eV belonging to V 2p_3/2_ and V 2p_1/2_, respectively, of the V^3+^ oxidation state. (1-2) The asymmetric peak at 529.99 eV is related to O 1s of the metal oxide onions. The Cr 2p XPS spectrum in Figure 5C displays two peaks at 576.49 and 586.19 eV related to Cr 2p_3/2_ and Cr 2p_1/2_, respectively, for the Cr^3+^ oxidation state. (3). The Zn 2p XPS spectrum in Figure 5D has two sharp peaks at 1021.2 Ev and 1044.2 Ev belonging to Zn 2p_3/2_ and Zn 2p_1/2_, respectively, for the Zn^2+^ oxidation state. (1-3) The XPS result confirms the purity and the chemical formula of the prepared NPs as Zn^2+^(VCr)^6+^O_4_.

### 3.2 Antibacterial studies

#### 3.2.1 MIC/MBC

**FIGURE 6:**
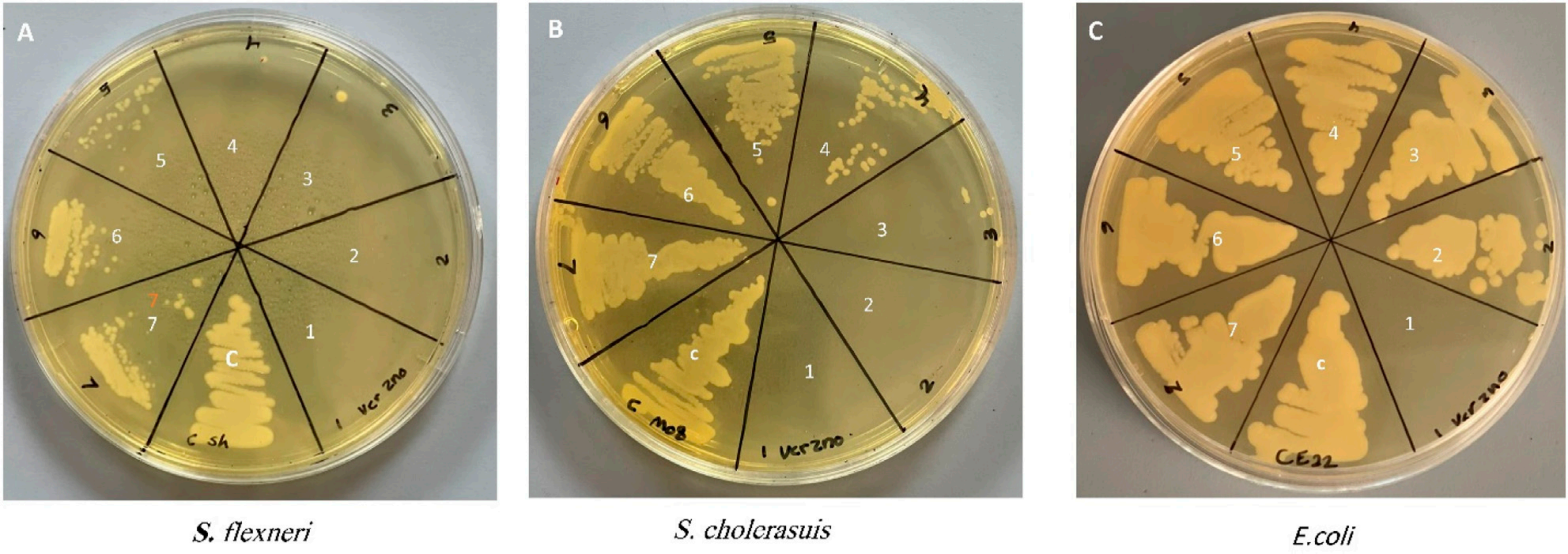
Photo depicting the Minimum Inhibitory Concentration (MIC) and Minimum Bactericidal Concentration (MBC) of the tested bacteria. Each number on photo represents a different concentration as follows: (1) 16 mg/mL, (2) 8 mg/mL, (3) 4 mg/mL, (4) 2 mg/mL, (5) 1 mg/mL, (6) 0.5 mg/mL, (7) 0.25: mg/mL, **(C)** control group of untreated bacteria.

Determination of the antibacterial activity of Zinc Chromium vanadate VCrZnO4 through the broth dilution method obtained the growth inhibition of all the bacteria tested. The antibacterial activities were examined using three gram-negative bacteria (*S. flexneri*, *S. cholerasuis*, and *E. coli*). Bacteria were treated with different concentrations of VCrZnO4. It was observed that with increasing nanomaterial concentration, the MIC/MBC was reduced, indicating the effect of nanomaterial on the tested bacteria. As shown in Figure 6, the MIC/MBC for *S. flexneri*, *S. cholerasuis*, and *E. col* was 1/2, 2/4, and 16/16 mg/mL, respectively. This indicated variation in the inhibitory effect. The presented results summarize the effect on bacteria growth when treated with VCrZnO4 compared to the untreated bacteria grown under the same conditions on the gram-negative bacteria. However, *E. col*i had a higher MBC and MBC than *S. flexneri and S. cholerasuis*. The nanomaterial overall showed the inhibition of growth with all three gram-negative bacteria, mostly water-dwelling organisms responsible for many enteric infections.

The remarkable versatility of nanoparticles (NPs) as antibacterial agents cannot be overstated. These tiny warriors have been shown to combat a wide range of Gram-positive and Gram-negative bacteria with unparalleled efficiency. For example, previously, ZnO NPs have been observed wielding their antimicrobial prowess against *Staphylococcus aureus*. At the same time, the valiant Ag NPs demonstrate a concentration-dependent capacity to vanquish foes such as *Escherichia coli* and *Pseudomonas aeruginosa* (Ramalingam et al., 2016)*.* The remarkable antimicrobial properties of these microscopic wonders are a testament to modern science and technology.

Several studies have assessed the antibacterial properties of ZnO-Nps using the broth dilution method against a range of bacterial strains, including *Pseudomonas aeruginosa*, *Staphylococcus aureus*, *Escherichia coli*, and *Klebsiella pneumoniae* (Yamamoto, 2001; Raghupathi et al., 2011; Sirelkhatim et al., 2015; Ramalingam et al., 2016; Fakhroueian et al., 2019). The observed results indicate that direct contact of ZnO-NPs with cell walls leads to the destruction of bacterial cell integrity (Adams et al., 2006; Brayner et al., 2006; Zhang et al., 2007; Shu et al., 2017), release of antimicrobial ions primarily Zn2+ ions (Kasemets et al., 2009; Li et al., 2011), and the formation of ROS (Sawai et al., 1998; Zhang et al., 2008; Jalal et al., 2010). The antibacterial activity of nanoparticles has been attributed to their unique structural and chemical properties, including its high surface area, porous structure, and metal ions that can interact with bacterial cell membranes and disrupt their function. The exact mechanism of the antibacterial action of nanoparticles is still under investigation. However, these findings suggest these nanoparticles have potential as a new antibacterial agent and could be further explored for their applications in medicine and healthcare.

#### 3.2.2 Morphogenesis of treated bacterial cells

**FIGURE 7:**
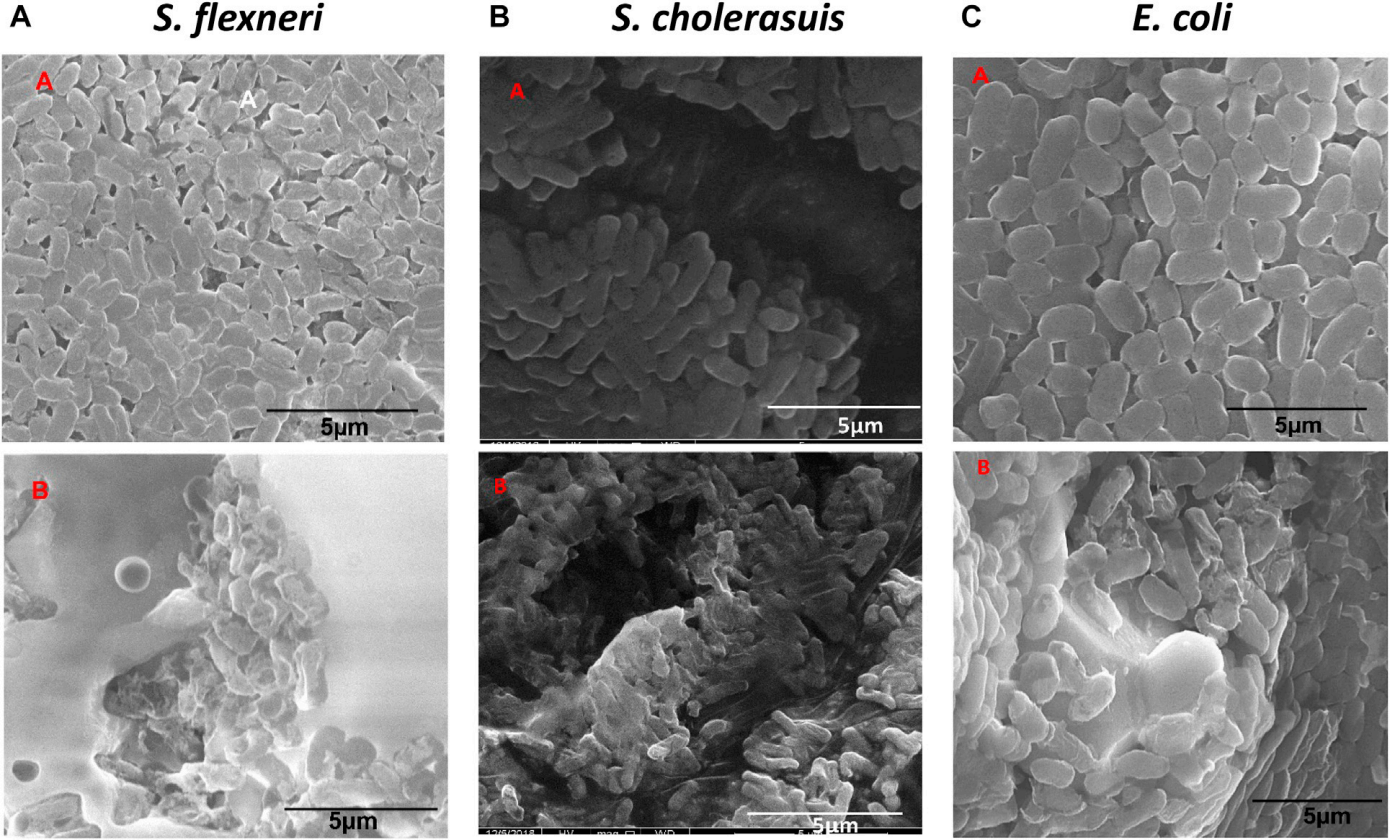
Morphological changes in three bacterial cells following treatment with nanoparticles VCrZnO_4_ nanoparticles. **(A)** Control group of untreated cells. **(B)** Cells treated with VCrZnO4 nanoparticles at the selected minimum inhibitory concentration (MIC).

The concentration chosen for this study was the MIC concentration of the nanomaterial for each strain. With the aid of a control experiment, i.e., untreated bacteria, the treated *S. flexneri*, *S. cholerasuis*, and *E. coli* showed the most abnormal appearance, as seen in Figure 7. The control cells displayed normal shape with intact cell surfaces (Figure 7A.). The bacteria displayed deformations ranging from mild to severe after being treated with Zinc Chromium vanadate VCrZnO4. Observations made in Figure 7B exhibit a remarkable distortion of *S. flexneri* cells, including severe disruption of the cell membrane. *S. cholerasuis* and *E. coli* also displayed comparable aberrations, where the once regular rod-shaped morphology was significantly compromised, manifesting in irregular shape and size, coupled with marked damage to the cellular surface. A possible mechanism for inhibiting bacterial proliferation is the attachment of nanomaterial to the bacterial surface after it encounters the nanomaterial (Shu et al., 2017). By attaching nanomaterial to the surface of the bacterial cell, membrane integrity is compromised, ultimately leading to cell death. Reactive oxygen species (ROS) generated by nanomaterial attachment and bacterial cells are also considered crucial in bacterial cell morphogenesis.

Several studies have shown that when bacteria are treated with zinc oxide nanoparticles, it can cause damage to their cell walls and membranes, leading to changes in their morphology and, ultimately, cell death. For instance, a study by Li et al. (2010) investigated the effect of zinc oxide nanoparticles on the growth of *Escherichia coli* bacteria (Li et al., 2011). The researchers found that even at low concentrations, zinc oxide nanoparticles could inhibit the growth of the bacteria, and at higher concentrations, it caused significant damage to the bacterial cells, including changes in their shape and size. While Zinc Chromium vanadate s has shown promising results as an antimicrobial agent, it is important to note that its use may negatively affect other organisms, including beneficial bacteria and humans. The Zinc Chromium vanadate nanoparticles could be toxic to certain types of beneficial bacteria found in the gut, potentially leading to disruptions in the microbiome and associated health problems (Yoo et al., 2021). Therefore, while Zinc Chromium vanadate shows promise as an antimicrobial agent, further research is needed to fully understand its potential benefits and risks, as well as its mechanisms of action and potential applications in medicine and industry.

### 3.3 Anticancer assay

#### 3.3.1 Cell viability by MTT assay

**FIGURE 8:**
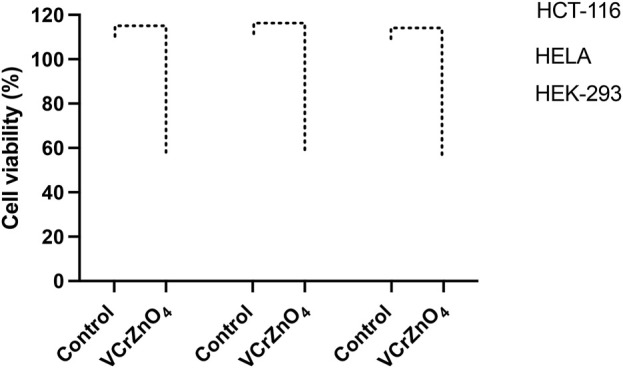
Figure 8: Impact of VCrZnO4 nanoparticle treatment on the viability of HCT-116, HELA, and HEK-293 cells after 48 h of treatment. The graph depicts the observed effects on cell viability. ****p < 0.0001.

The impact of nanoparticles VCrZnO_4_ on HCT-116 and HELA cells was examined. Post 48 h; we found a significant decrease in cancer cells post-treatment of nanoparticles VCrZnO4 in both HCT-116 and HELA cells (Figure 8 *)*. While the treatment of nanoparticles VCrZnO showed better and stronger inhibitory action on HELA cells than on HCT-116 cells (Figure 8). The treatment of nanoparticles VCrZnO4 on HEK-293 cells showed a decline in the normal cell population. However, the treatment of nanoparticles produced less cytotoxicity on normal HEK-293 cells than on HCT-116 and HeLa cells (Figure 8).

Our results suggest that VCrZnO4 nanoparticles exhibit significant cytotoxicity against HCT-116 and HELA cells. Many studies have shown that nanomaterials and biomaterials produce anticancer activities. For example, ZnO nanoparticles showed a dynamic cytotoxic effect in cervical carcinoma cells, and the impact of ZnO nanoparticles on cancer cells was due to the induction of apoptosis through increased intracellular ROS levels (Pandurangan et al., 2016). In another study, the effect of ZnO nanoparticles was studied against small-cell lung cancer where ZnO nanoparticles induced cancer cell death by activating reactive oxygen species and DNA leakage from nuclei and also observed the upregulation of apoptotic gene expression (Tanino et al., 2020). In another study, thymoquinone-conjugated ZnO nanoparticles mediated cytotoxicity and anticancer action on breast cancer cells (Banupriya et al., 2022). ZnO nanoparticles conjugated with N-succinyl chitosan showed strong anticancer activities on breast cancer cells (MDA-MB-231) by inducing apoptotic action (Ghaffari et al., 2020). The treatment of ZnO nanoparticles containing Oxaliplatin via polymerization showed strong anticancer action on colorectal cancer cells, as examined in both *in vitro* studies and *in vivo* mouse models (Alavi et al., 2023). In addition, the treatment of pure ZnO nanoparticles and doped with molybdenum and graphene oxide nanoparticles showed anticancer activity on the human colon (HCT116) and breast (MCF7) cancer cells, and anticancer action was mediated through reactive oxygen species, p53, and the caspase-3 pathway (Ahamed et al., 2022). Applying chromium oxide (CrO) nanoparticles also exhibited anticancer activity on MCF-7 cells, HepG2, and HUH-7 cancer cells (Jermy et al., 2019; Li et al., 2020). Besides, there have been numerous other studies that have shown that both nanomaterials and biomaterials can produce anticancer activities. These materials can be used in various ways to help prevent, diagnose, and treat cancer, and they have shown promising results in preclinical and clinical studies (Connor et al., 2005). Researchers used gold nanoparticles to target and destroy tumor cells in mice (Peng et al., 2019). The result underpins that the nanoparticles could deliver a drug directly to the tumor cells, significantly reducing tumor growth and increasing survival rates in the mice (Peng et al., 2019). The use of hydrogel nanoparticles could be used to deliver a drug directly to the tumor cells, resulting in significantly reduced tumor growth and increased survival rates in mice (Biondi et al., 2015; Nolan et al., 2018; Rafieian et al., 2019; Jiang et al., 2020; Nadtoka et al., 2020; Stojkovska et al., 2020; Clasky et al., 2021; Sulaiman et al., 2021; Nunes et al., 2022).

#### 3.3.2 Apoptotic death of cancer cells as revealed by DAPI staining

**FIGURE 9:**
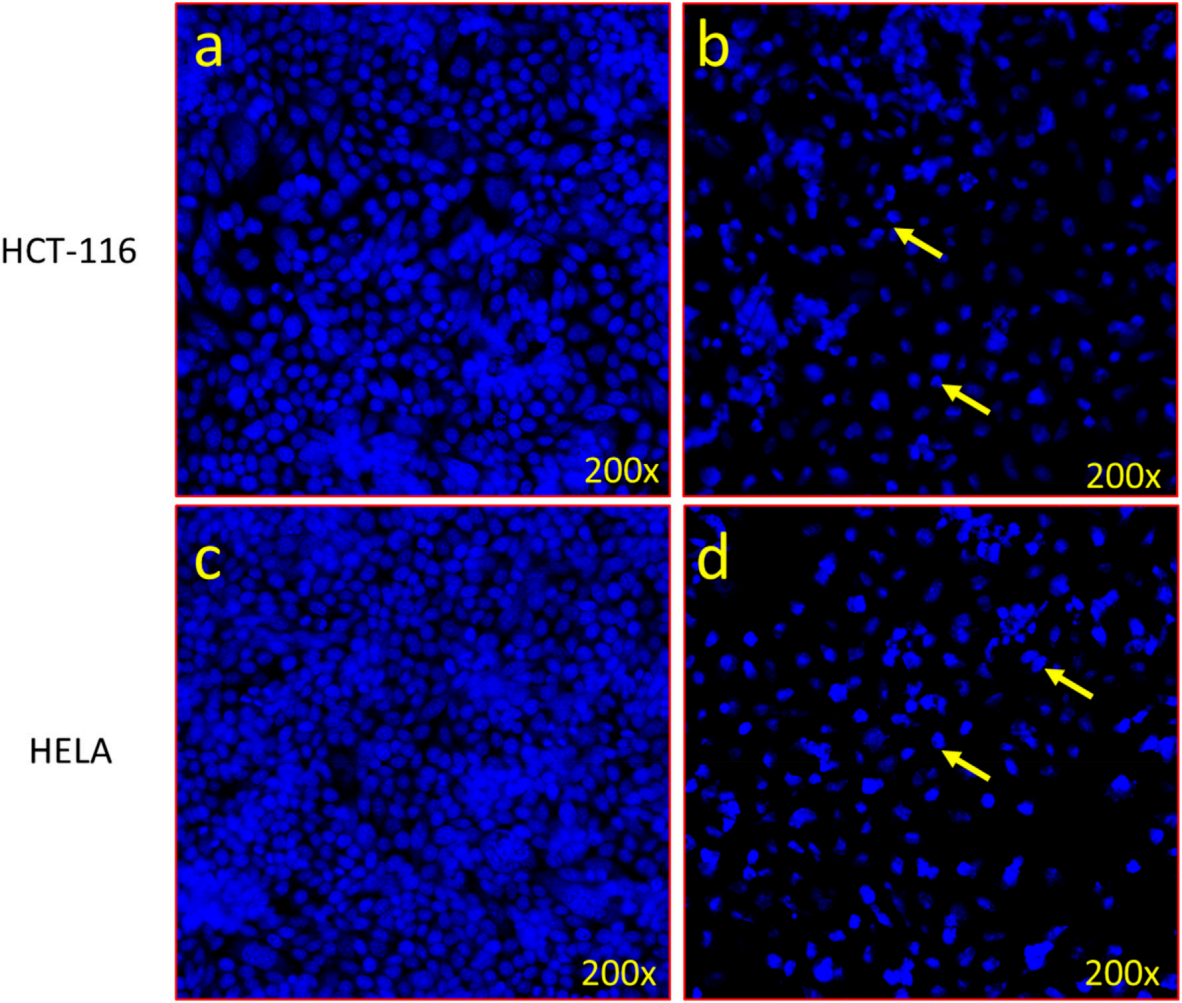
Apoptotic death of cancer cells revealed by DAPI staining. **(A)** Control cells with normal morphology and intact, healthy cells. **(B)** Impact of VCrZnO4 nanoparticles on colon cancer cells (HCT-116) after 48 h of treatment (60 μg/mL). **(C)** Control cells with normal morphology and intact, healthy cells. **(D)** Impact of VCrZnO4 nanoparticles on HELA cancer cells. Arrows indicate observed chromatin condensation, nuclear augmentation, and formation of apoptotic bodies.

In order to better understand the cause of cancer cell death, we examined both HCT-116 and HELA cells using DAPI (4′,6-diamidino-2-phenylindole), a blue-fluorescent DNA stain used to identify apoptosis. Figures 9A, B depict that administering VCrZnO4 NPs decreases HCT-116 colon cancer cell death. Similarly; Figures 9B, D displayed a reduction in cell death for HELA cervical cancer cells. Notably, post-treatment with VCrZnO4 nanoparticles, we observed chromatin condensation and the presence of apoptotic bodies in HCT-166 cells (Figures 9B,D). In contract the nuclei in the control group exhibited no discernible structural or aesthetic changes (Figures 9A,C).

In Figure 9B, VCrZnO4 nanoparticles are shown in response to colon cancer cells (HCT-116), with Figure 9A representing the control cells with normal morphology and intact and healthy cells; and 8b representing the responses after 48 h of treatment (60 ug/mL). Figures 9D–F show the impact of nanoparticles VCrZnO4 on HELA cancer cells, with Figure 9B depicting the control cells with normal morphology and intact structure. Arrows in the figures highlight observed phenomena such as chromatin condensation, nuclear augmentation, and the formation of apoptotic bodies. In addition, IC_50_ values have also been calculated and are shown in Table 1.

**TABLE 1:** Calculation of IC_50_ after treatment of VCrZnO_4_.

	IC_50_ µg/mL		
	HCT-116	HELA	HEK-293
VCrZnO4	38.50 + 3.50	42.25 + 4.15	43.55 + 4.85

Of note, nanoparticles have also shown promising potential in inducing apoptotic death of cancer cells. Apoptosis induction in cancer cells is a promising approach for cancer therapy as it selectively targets and eliminates cancer cells while sparing normal cells. Many studies suggest nanocomposites as a promising approach to kill cancer cells. Gold nanoparticles decorated with polypyrrole induced apoptotic death in breast cancer cells. The study investigated the potential use of polypyrrole-coated gold nanoparticles as a photothermal therapy for breast cancer cells (Sun et al., 2018). The researchers found that the nanoparticles could selectively target breast cancer cells and induce apoptotic cell death upon exposure to near-infrared laser irradiation. The study suggested that polypyrrole-coated gold nanoparticles could be a promising and effective therapy for breast cancer. Further, zinc oxide nanoparticles also induced apoptotic death in prostate cancer cells by activating the caspase pathway. In addition, silver nanoparticles coated with graphene oxide or reduced graphene oxide could be a potential therapeutic agent for lung cancer (Yadav et al., 2022). These results have shown promising potential in inducing apoptosis of cancer cells, which could be a selective and practical approach for cancer therapy. Further research is needed to evaluate their safety and efficacy in clinical settings.

## 4 Conclusion

This study successfully synthesized VCrZnO4 nanoparticles and investigated their antibacterial and anticancer properties. The comprehensive analysis using various characterization techniques provided insights into the structural and morphological characteristics of the nanomaterials. While the synthesized nanoparticles showed potential as a dual solution for eradicating waterborne*Enterobacteriaceae* and fighting cancer, further research is needed to confirm their effectiveness in treating bacterial infections and cancers through *in vivo* studies. Moreover, it would be valuable for future investigations to explore their potential in areas like drug delivery and wound healing, assessing long-term safety and effectiveness, and evaluating their efficacy against cancer cells. These findings contribute to the advancement of nanomedicine and highlight the potential of VCrZnO4 nanoparticles as promising candidates for antibacterial and anticancer applications, paving the way for developing more effective therapeutic strategies.

## 5 Possible limitations of the study

While the study on Zinc Chromium vanadate nanoparticles (VCrZnO4 NPs) holds promise for potential antibacterial and anticancer applications, it is important to acknowledge several limitations. Firstly, the study was conducted solely *in vitro*, highlighting the need for further research to assess the efficacy of the NPs *in vivo*. Secondly, the study only tested the NPs against a limited number of bacterial strains and cancer cells. Additional testing against a broader range of pathogens and cancer cell lines is necessary to confirm the general applicability of the NPs. Thirdly, the long-term effects of exposure to the NPs are still unknown, and future studies need to explore the potential toxicity of VCrZnO4 NPs on human cells. Finally, the synthesis of VCrZnO4 NPs using a one-pot solvothermal method may be challenging for large-scale production, limiting the nanomaterials’ practical applications. Addressing these limitations through further research will be instrumental in realizing the full potential of VCrZnO4 Nps as versatile nanomaterials for antibacterial and anticancer therapies.

## Data Availability

The raw data supporting the conclusion of this article will be made available by the authors, without undue reservation.
